# The role of male scent in female attraction in the bank vole, *Myodes glareolus*

**DOI:** 10.1038/s41598-024-55235-x

**Published:** 2024-02-27

**Authors:** Holly A. Coombes, Mark C. Prescott, Paula Stockley, Robert J. Beynon, Jane L. Hurst

**Affiliations:** 1 Mammalian Behaviour and Evolution Group, Institute of Infection, Veterinary and Ecological Sciences, University of Liverpool, Liverpool, UK; 2 Department of Biology, University of Oxford, Oxford, UK; 3 Centre for Proteome Research, Institute of Systems, Molecular and Integrative Biology, University of Liverpool, Liverpool, UK

**Keywords:** Animal behaviour, Zoology

## Abstract

Chemical signals are frequently utilised by male mammals for intersexual communication and females are often attracted to male scent. However, the mechanism underlying female attraction has only been identified in a small number of mammalian species. Mammalian scents contain airborne volatiles, that are detected by receivers at a distance from the scent source, as well as non-volatile molecules, such as proteins, that require physical contact for detection. Lipocalin proteins, produced within the scent secretions of many terrestrial mammals, are thought to be particularly important in chemical signalling. Here, we explore if the male-specific protein, glareosin, expressed by adult male bank voles, *Myodes glareolus*, stimulates female attraction to male scent. We show that female bank voles are more attracted to male compared to female scent, supporting the results of previous studies. Increased investigation and attraction to male scent occurred to both airborne volatiles and non-volatile proteins when they were presented separately. However, we found no evidence that attraction to male scent was driven by glareosin. Our results differ from those previously described in house mice, where a single protein induces female attraction to male scent, suggesting the mechanism underlying female attraction to male scent differs between species.

## Introduction

Male mammals often invest heavily in species-specific chemical signals that are used by females to locate, assess, and recognise high quality mates^1^. In terrestrial mammals, males typically broadcast chemical information to females via scent marks, which they deposit around their home area^2^. Female attraction to male scent marks is normally initiated by airborne volatile organic compounds (VOCs) emitted from the scent source that can be detected at distance^3^. Upon detection of airborne volatiles, individuals typically approach and further investigate the scent source^4^. Non-volatile components of the scent, such as proteins and peptides, together with VOCs within the scent source, provide further information following nasal or oral contact^5–8^. As well as providing additional information about the signaller, many scent communication proteins bind smaller VOCs^9^ and slow their release from scent deposits^10^ allowing males to advertise to females long after scent marks were initially deposited^11^.

Rodents often exhibit proteinuria, excreting a high concentration of species-specific proteins within their urinary scent marks^12,13^. Many of these communication proteins are from the lipocalin family and can be separated into two main groups, major urinary proteins (MUPs), predominately excreted in the urine of mice and rats^12,13^, and odorant binding proteins (OBPs)^14^. While mammalian OBPs are most commonly found in the mucus of the nasal cavity, where they are presumed to play a role in the binding and detection of volatile pheromones, some OBPs and MUPs can be expressed at high level in scent secretions while others are expressed in association with olfactory tissues^14^. This suggests a similar dual function within both groups, with some isoforms involved in scent detection and others in scent signalling^14^. Proteins in scent secretions from several rodent species have been identified and sequenced, but assessment of their function through behavioural tests has been attempted in only a few species^15–17^. The best studied example of a urinary protein that mediates female attraction to male scent in rodents is in the house mouse, *Mus musculus*^16^. Male house mice express male-specific high levels of a MUP called darcin (MUP20) that stimulates female attraction to male urine^18^. This MUP sex pheromone also stimulates a learned attraction to both the male’s individual scent signature and remembered attraction to the location where females encountered the darcin pheromone^19,20^. This attracts females to the location and odour profile of male territory owners, assessed through rates of scent marking and countermarking, when females are ready to mate^16^.

By contrast, voles express urinary proteins belonging to the OBP family^21–23^. Male bank voles, *Myodes glareolus,* produce a high concentration of urinary protein that is androgen-dependent^24^. The majority of their urinary protein output comprises a 16,930 Da male-specific OBP named glareosin^22^, although several other OBPs expressed at much lower levels have also been identified^23^. Glareosin is expressed only by adult males during the breeding season, suggesting a likely function in male sexual and/or competitive communication^22^. A similar protein was identified from water vole urine in which there is also male-biased expression^21^. Less is known about the urinary VOC profile of bank voles but studies in related species have found differences in urinary volatiles on the basis of sex^25^, season^21^ and male breeding condition^26^.

Although the urinary proteins of bank voles have been relatively well described, little is known about the biological function of these proteins. Male bank voles exhibit sexually dimorphic urinary scent marking^27^, suggesting male chemical signalling is important in this species. Female bank voles can discriminate between urine from different males based on social status^28–30^, familiarity^31^ and castration^32^. However, few have explored which components in male scent signals facilitate female attraction to male scents and their ability to discriminate between potential mates.

Here, we explore whether the male-specific protein glareosin in bank vole urine facilitates female attraction to conspecific male scents. We hypothesised that glareosin would induce female attraction to male scents via direct contact with male urine through a similar mechanism underlying female house mouse response to the male protein pheromone darcin (MUP20)^16^. In a series of behavioural assays, we compared female responses to unfamiliar male and female scents with and without nasal contact to explore the potential role of male chemical signals in sexual attraction and test whether nasal contact with scents is required to mediate female attraction. After confirming that females were attracted to spend more time with male compared to female urine, urine was fractionated into a low molecular weight (LMW, < 3 kDa) and high molecular weight fraction (HMW). We then tested whether females exhibited greater sexual attraction to the high compared to the low molecular weight fraction. Lastly, we then manipulated male scents to determine the potential importance of glareosin in eliciting female attraction to male urine. Behavioural tests were carried out in females’ home enclosures to model the natural situation where they occupy exclusive home ranges overlapped by multiple males^33^. We used the time spent sniffing a stimulus as an indication of female motivation to gain information from the scent, and the time near the stimulus when not sniffing as a measure of prolonged female attraction to the scent.

## Results

### Females are attracted to male urine

**Figure 1:**
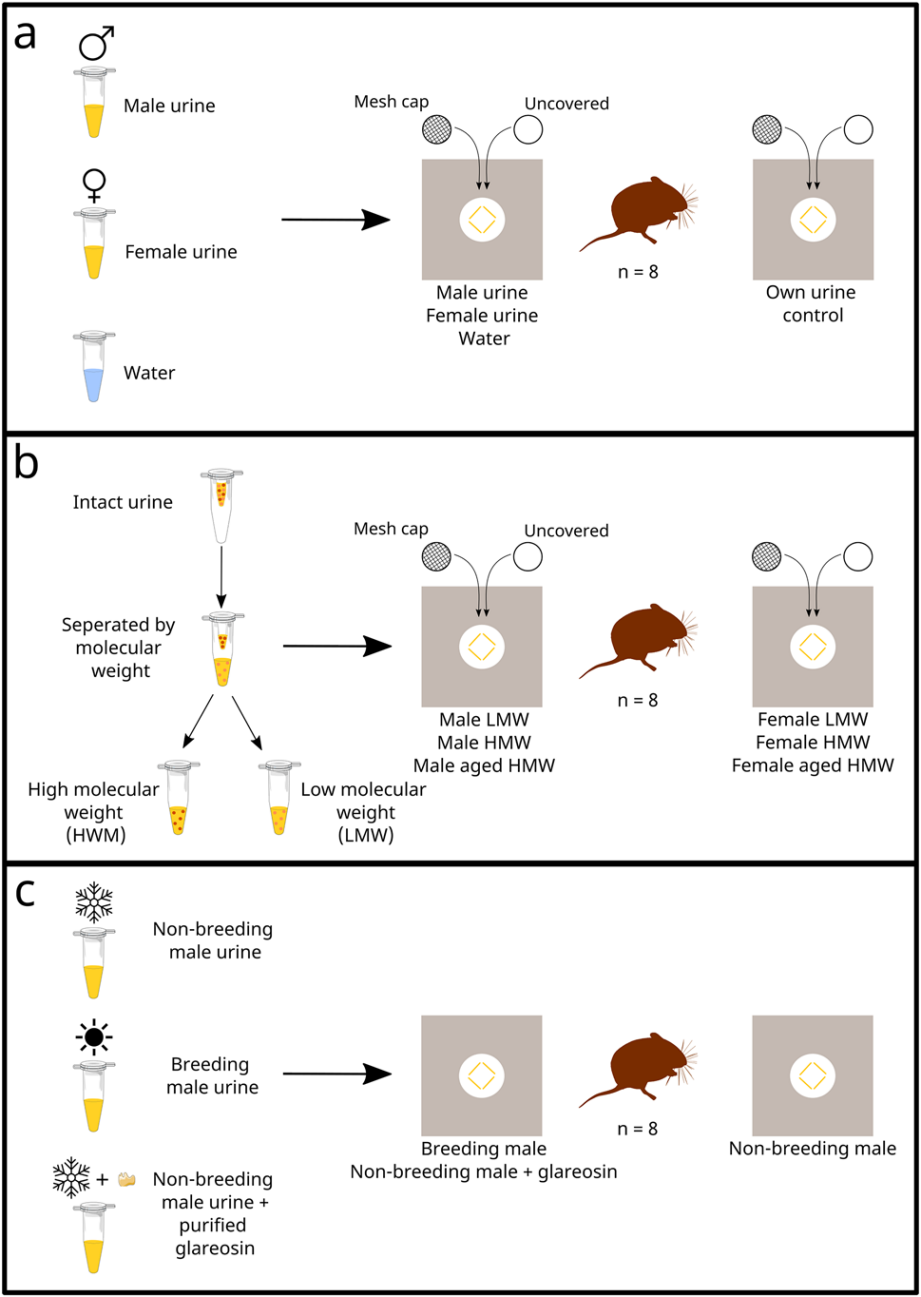
Overview of experimental design. (**a**) Females were tested with their own urine presented alongside either conspecific male urine, female urine or water. Matched stimuli were presented either covered by mesh caps to prevent stimulus contact or uncovered. (**b**) Urine collected from male and female conspecifics was fractionated by molecular weight (LMW: passed through 3 kDa filter; HMW: retained by 3 kDa filter) before testing females with equivalent male and female fractions, presented at the same time. (**c**) Females were tested with non-breeding male urine presented alongside either breeding male urine or urine from non-breeding males supplemented with glareosin. Created using Inkscape, with specific icons: microtube-closed-translucent by Servier (https://smart.servier.com/) is licensed under CC-BY 3.0 (https://creativecommons.org/licenses/by/3.0/), Myodes glareolus by Callum Le Lay (https://www.phylopic.org/images/f7d6d04c-73fa-4bf3-8c94-48134e6857b9/myodes-glareolus) is licensed under CC0 1.0 (https://creativecommons.org/publicdomain/zero/1.0/).

To confirm that female bank voles are attracted to scents from conspecific adult males, we compared the response of resident females to urine from unfamiliar conspecific males or females, or to water introduced into their individual home enclosures. In each test, own urine was presented alongside the test stimulus as a control (Fig. 1a). We calculated the bias in time spent sniffing the test stimulus minus their own urine control, or time nearby but not sniffing, as measures of female attraction. We also investigated whether nasal contact with scent components influenced female attraction by presenting each of the three test stimuli, and the matched own urine control, either uncovered or covered by a mesh cap that allowed detection of airborne volatiles only.

**Figure 2:**
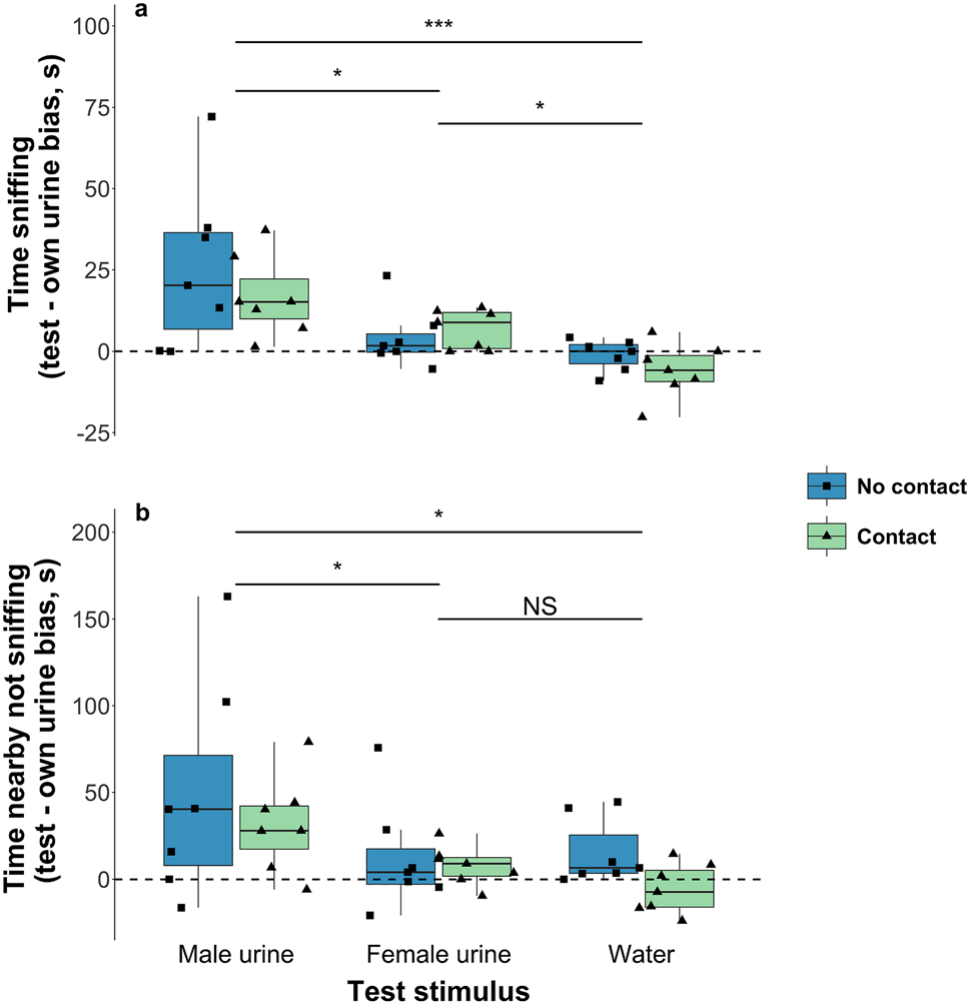
Females are attracted to male urine, regardless of ability to contact the scent. Effect of stimulus type and contact on the time females (n = 8) spent sniffing (**a**), or nearby not sniffing (**b**) conspecific urine or water stimuli relative to an own urine control stimulus, presented simultaneously. Values that fall below 0 (dashed black line) indicate that females spent more time nearby or sniffing their own urine, values that fall above 0 indicate that females spent more time nearby or sniffing the test stimulus. Horizontal black lines within bars indicate median values, boxes represent the interquartile range, whiskers represent 1.5 × the interquartile range; triangles (contact) and squares (no contact) show individual data points. Statistical significance of planned contrasts or Tukey post-hoc tests between different test stimuli (see text): ***p < 0.001, *p < 0.05, NS p > 0.05.

Females were attracted to conspecific male urine (Fig. 2). Females differed in response to the three test stimuli, both with respect to time actively sniffing the stimuli (LMM, χ^2^ = 27.40, 2 df, p < 0.001) and time nearby but not sniffing (LMM, χ^2^ = 7.61, 2 df, p = 0.022). Planned contrasts confirmed that females spent significantly more time with male urine than with either water (sniffing: t_1_ = 4.91, p < 0.001; nearby not sniffing: t_1_ = 2.37, p = 0.025) or unfamiliar female urine (sniffing: t_1_ = 2.09, p = 0.045; nearby not sniffing: t_1_ = 2.34, p = 0.026). Bias scores, calculated by subtracting time nearby or sniffing own urine from time nearby or sniffing the test stimulus, were on average four times greater towards male than female urine (mean bias in time nearby male urine = 40.4 ± 12.7 s, female urine = 10.2 ± 6.1 s, water = 5.0 ± 5.2 s; mean bias in time sniffing male urine = 21.2 ± 5.3 s, female urine = 5.6 ± 2.0 s, water = − 2.35 ± 1.9 s). Although female voles investigated urine from an unfamiliar female more than a water control (Tukey post-hoc, t_1_ = 2.80, p = 0.024), they did not spend more time near unfamiliar female urine compared to water while not actively sniffing (Tukey post-hoc, t_1_ = 0.007, p = 1.00). Thus, females spent time gaining information from an unfamiliar female scent, but were not attracted to spend more time near this stimulus in contrast to their attraction to male urine.

Notably, allowing females to gain only airborne information from scents through a mesh barrier had no effect on either the time they spent actively sniffing stimuli (LMM, χ^2^ = 0.22, 1 df, p = 0.64) or nearby the stimuli without sniffing (LMM, χ^2^ = 0.035, 1 df, p = 0.85). There was also no significant interaction between the ability to contact the stimulus and stimulus type (Fig. 2, AIC of time sniffing model with interaction = 148.26, without interaction = 148.03; AIC of time nearby model with interaction = 101.45, without interaction = 101.17).

### Females are attracted to male urinary volatiles

**Figure 3:**
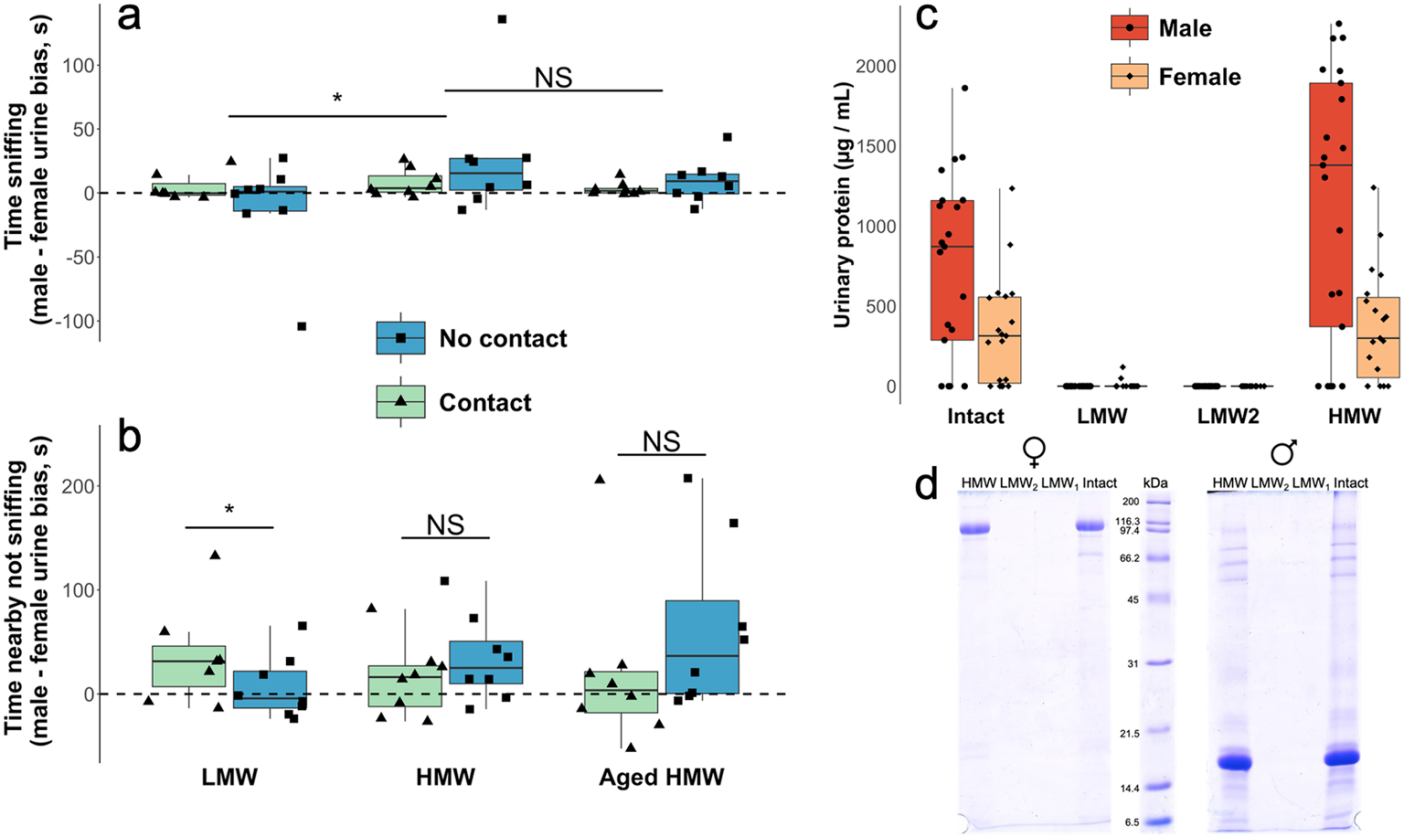
Female attraction to low and high molecular weight fractions of male urine compared to female urine fractions**.** The amount of time female voles (n = 8) spent sniffing (**a**) and nearby (**b**) low molecular weight (LMW), high molecular weight (HMW) or 5-day aged HMW (Aged HMW) urinary fractions when they were able to contact the scent or when contact was prevented. Values that fall above 0 (dashed black line) indicate more time with male urinary fractions, values that fall below 0 indicate more time with female fractions. Horizontal black lines indicate median values, boxes represent the interquartile range, whiskers represent 1.5 × the interquartile range; triangles (contact) and squares (no contact) show individual data points. Successful fractionation of urine was confirmed by protein assays to check total protein concentration (**c**) and SDS PAGE to check protein composition (**d**) of both intact and fractionated urine. LMW2 relates to a second LMW fraction collected during urinary fractionation that was not used in any behavioural tests. See Supplementary Figs. S7 and S8 for original example gel image. Statistical significance of LMM (**a**) and planned contrasts (**b**) between different test stimuli (see text): ***p < 0.001, *p < 0.05, NS p > 0.05.

To explore the importance of volatile and non-volatile urinary components in stimulating female attraction, we separated male and female urine from unfamiliar donors into high molecular weight (HMW) and low molecular weight (LMW) fractions by centrifugation through a 3 kDa filter. The LMW fraction comprised low molecular weight molecules that passed through the filter (< 3 kDa), including salts, metabolites, small peptides, and other VOCs soluble in the aqueous urine, but no detectable proteins (Fig. 3c,d). The HMW fraction contained proteins (> 3 kDa, Fig. 3c,d) and any low molecular weight compounds that did not pass through the filter or were bound to proteins. As the HMW was passed through the concentrator twice, we estimate that the level of low molecular weight material not bound to proteins but remaining in the HMW fraction was less than 2% of that in intact urine. We compared female responses to each male fraction when presented simultaneously with the equivalent female fraction in the female’s home enclosure (Fig. 1b). Stimuli were again presented either uncovered, allowing full nasal contact, or covered by a mesh cap that gave access to airborne molecules only.

We first confirmed that females were more attracted to male compared to the equivalent female urinary fractions. As expected from the stronger attraction shown towards male intact urine, females spent more time sniffing the urine fractions of male donors (LMM, χ^2^ = 16.96, 1 df, p < 0.001), as well as more time nearby the male fractions when not actively investigating the stimulus (LMM, χ^2^ = 9.72, 1 df, p = 0.0018). Females spent more time investigating urine fractions from both male and female donors when they could contact the scent (LMM, χ^2^ = 22.09, 1 df, p < 0.001, mean time spent sniffing with contact = 26.2 ± 5.3 s, without contact = 8.9 ± 1.9 s), suggesting that females gained additional information from direct contact with the conspecific urinary fractions potentially due to detection of molecules via the VNO^15^. However, contact did not affect the amount of time females spent nearby conspecific fractions when not actively investigating (LMM, χ^2^ = 1.19, 1 df, p = 0.28).

To assess whether there was any difference in the strength of female preference for the male over the female stimulus between LMW versus HMW urine fractions, and whether ability to contact the scent influenced this, we calculated the bias in time spent sniffing or nearby the male fraction compared to the same female fraction. Females tended to investigate male HMW fractions more than LMW fractions (LMM, χ^2^ = 2.88, 1 df, p = 0.090, mean bias in sniffing male compared to female HMW fractions = 16.9 ± 8.5 s, LMW fractions = − 3.9 ± 7.8 s), regardless of their ability to contact the scent stimuli (LMM, no significant interaction between fraction and contact: χ^2^ = 0.32, 1 df, p = 0.57, Fig. 3a). As females were able to discriminate between the male and female HMW fraction based on airborne odours alone (i.e. without contact), this suggests that females detected VOCs emitted from the HMW fraction. Similar to the well-established role that MUPs play in binding VOCs in mouse urine, which are then slowly released from scent marks^10,34,35^, it is most likely that VOCs remained in the HMW fraction of male bank vole urine as ligands bound to urinary OBPs.

Contact appeared to have a stronger effect on time spent near male fractions when not actively sniffing the scent (LMM, interaction between fraction and contact: χ^2^ = 6.62, 1 df, p = 0.010). Females spent less time near the male LMW fraction when they could contact the stimulus (planned contrasts: t_1_ = 2.15, p = 0.044; Fig. 3b, mean bias towards male LMW fraction with contact = 6.6 ± 10.7 s, without contact = 36.7 ± 18.6 s). The LMW fraction mainly contains soluble VOCs which become airborne as they evaporate from the scent mark. Females are attracted by these airborne volatiles (see section “Females are attracted to male urine”), which induce them to approach and investigate non-volatile components in the scent through nasal contact with the source. Females may have lost interest in the LMW fraction on contact due to the absence of non-volatile molecules such as urinary proteins in this fraction. By contrast, the HMW fraction contained a substantial amount of urinary protein (Fig. 3c,d), with females showing sustained attraction on contact. They also continued to show strong attraction to the HMW fraction even when contact was prevented (planned contrasts comparing bias in time spent nearby male HMW with and without contact: t_1_ = 1.47, p = 0.16), choosing to spend time near the male scent in addition to investigation. This suggests that VOCs bound and released by urinary proteins in the HMW fraction were attractive to females regardless of any contact with the proteins themselves.

### Females are attracted to airborne ligands released from urinary proteins and to involatile urinary proteins on contact

To further explore the contribution of male urinary proteins and bound volatile ligands to female attraction, we streaked male and female HMW fractions onto filter paper and left these in the open for 5 days at ambient room temperature to allow any free VOCs and volatile ligands released from urinary proteins to evaporate. A preliminary analysis of urinary volatiles in bank voles by gas chromatography suggests that the majority of detectable volatiles are lost from intact male urine after 24 h (Figs. S1–S3). After 5 days, we expected that the majority of volatile components would be lost from the HMW fraction, unless tightly bound to urinary proteins. We then assessed female attraction to male versus female aged HMW urinary fractions presented with and without physical contact (Fig. 1b), and compared this to their response in the same test with fresh HMW.

We first confirmed that ageing of conspecific scent did not reduce female preference for male scent. Females still spent more time investigating male compared to female 5 day aged HMW fractions (LMM, χ^2^ = 20.52, 1 df, p < 0.001, mean time spent sniffing male fraction = 15.1 ± 4.7 s, female fraction = 8.60 ± 2.90 s) and spent more time nearby the male stimulus (LMM, χ^2^ = 8.42, 1 df, p = 0.0037, mean time nearby male fraction = 115 ± 23.1 s, female fraction = 73.4 ± 12.6 s). Direct contact increased female investigation of both male and female aged HMW fractions (LMM, χ^2^ = 22.37, 1 df, p < 0.001, mean time sniffing HMW fraction with contact = 62.9 ± 28.6 s, without contact = 20.4 ± 28.0 s), suggesting that both male and female HMW fractions contain non-volatile components of no or very low volatility that females detect upon contact. Females displayed reduced investigation of aged compared to fresh HMW fraction from both male and female donors (LMM, χ^2^ = 3.89, 1 df, p = 0.049, mean time sniffing fresh HMW fraction = 22.0 ± 5.9 s, aged HMW fraction = 11.8 ± 2.8 s). However, there was no significant effect of contact (LMM, χ^2^ = 1.89, 1 df, p = 0.17) or ageing (LMM, χ^2^ = 0.59, 1 df, p = 0.44) on the amount of time females spent with conspecific HMW fractions, suggesting they were still able to detect airborne volatiles given off from the fraction even after it had been aged for 5 days.

To assess if female preference for the male HMW fraction was reduced by ageing, we again calculated the bias towards the male compared to female fraction for time spent sniffing or nearby aged HMW fractions (Fig. 3a,b). Female preference for the male fraction did not depend upon the freshness of the HMW fraction (bias to male fraction LMM, sniffing: χ^2^ = 1.73, 1 df, p = 0.19, nearby: χ^2^ = 1.29, 1 df, p = 0.26). Further, there was no effect of contact on female bias towards the male HMW fraction (sniffing: χ^2^ = 1.046, 1 df, p = 0.31, nearby: χ^2^ = 1.53, 1 df, p = 0.22). Therefore, although female responses to male and female conspecific HMW fractions were stronger when the fractions were fresh and when they could directly contact the stimuli, female preference for the male over female fraction remained even when presented with aged HMW fractions they could not contact.

### Glareosin does not induce female attraction to urine from breeding males

Finally, as non-volatile components in male urine are strongly attractive to female bank voles upon contact, we tested whether the OBP glareosin induces female attraction. Glareosin is the predominant protein in male bank vole urine, showing species, sex and season-specific expression. Male, but not female, bank voles produce substantial quantities of glareosin in their urine during the breeding season (1.76 ± 0.27 µg/µL total urinary protein), declining to undetectable levels in bank voles captured during winter^22^. We purified glareosin (together with any bound ligands) from the urine of captive male bank voles maintained under a breeding season light cycle and temperature. Females were presented with male urine collected during the non-breeding season versus non-breeding male urine spiked with 1.5 µg/µL of glareosin (Fig. 1c). We also tested female preference between urine collected from wild non-breeding season males versus urine from captive donors kept under breeding season conditions to check that females were attracted to breeding season male urine.

**Figure 4:**
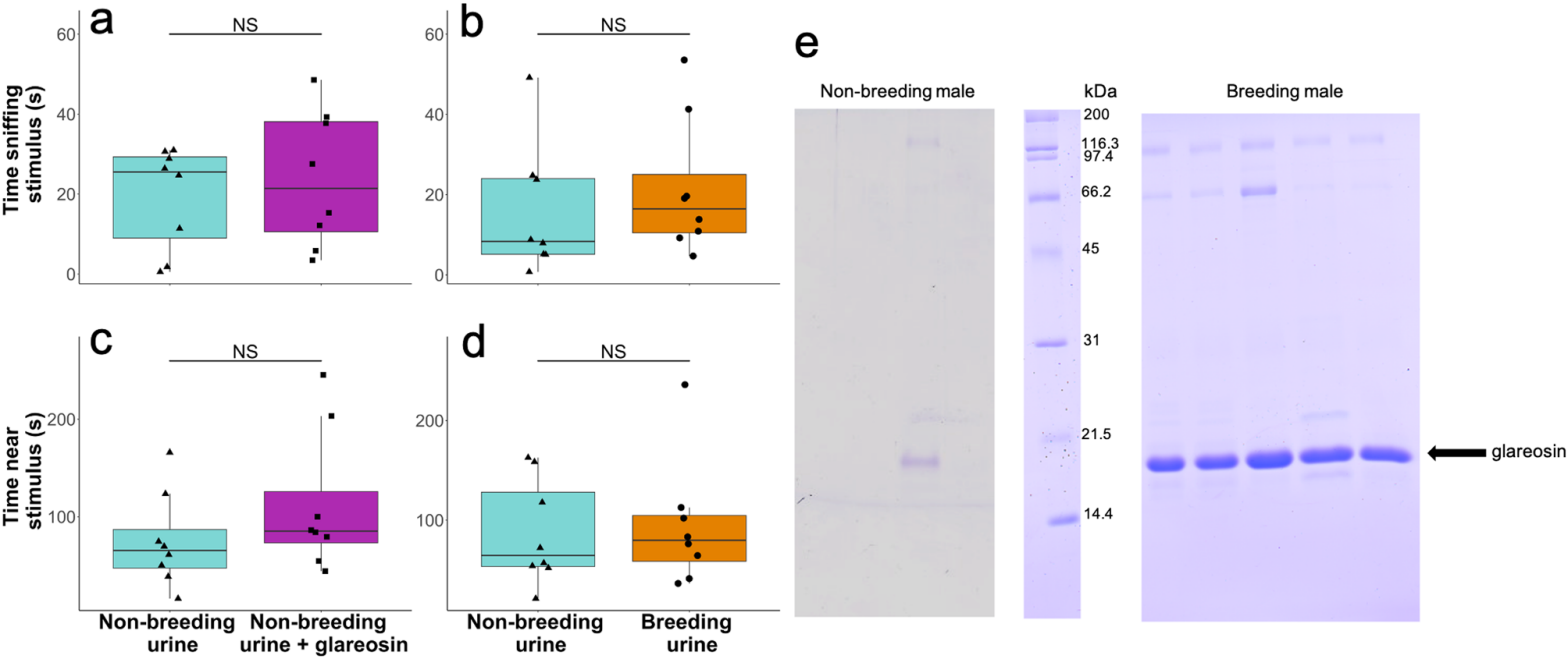
Glareosin did not increase female attraction when added to urine from non-breeding season males. The addition of glareosin to urine collected from males during the non-breeding season did not increase female (n = 8) investigation (**a**) or time spent nearby (**c**). There was no effect of season on female investigation of (**b**) and time spent nearby (**d**) male urine. Horizontal black lines within boxes indicate median values, boxes represent the interquartile range, whiskers represent 1.5 × the interquartile range, and black triangles (non-breeding urine), black squares (non-breeding urine + glareosin) and black circles (breeding urine) show individual data points. Statistical significance from LMM between different test stimuli (see text): NS p > 0.05. SDS-PAGE showing protein profile of male voles housed under breeding season conditions (breeding males) compared to non-breeding season males (**e**). See Supplementary Figs. S9–S10 for original gel images.

The addition of glareosin to male urine collected during the non-breeding season did not increase female attraction to the urine, with females spending no more time sniffing (t_7_ = 0.61, p = 0.56, Fig. 4a) or nearby the scent spiked with glareosin  (t_7_ = 1.05, p = 0.33, Fig. 4c). Surprisingly, females did not prefer urine collected from males held under breeding season compared to non-breeding season conditions (time sniffing: t_7_ = 0.92, p = 0.39, Fig. 4b; nearby not sniffing: t_7_ = 0.25, p = 0.81, Fig. 4d). We confirmed that captive males housed under breeding conditions expressed glareosin in their urine (Fig. 4e). By contrast wild males captured during the non-breeding season had much lower levels of glareosin expression (Fig. 4e), although a faint band can be seen in at least one of the non-breeding male donors suggesting some glareosin production occurred during the non-breeding season.

## Discussion

This study explores whether the male-specific urinary protein, glareosin, stimulates female attraction to male scent in bank voles. Although we found no evidence that female attraction was mediated by glareosin, females responded strongly to both low molecular weight components and the protein component of male urine when these were presented separately. Interestingly, preventing females from contacting the stimuli had little impact on female preference for male over female scents, suggesting that female attraction can be mediated by airborne volatiles alone. These results differ from those described in the well-studied house mouse system, suggesting the mechanism of scent-mediate female attraction may not be universal across rodents.

Female bank voles strongly preferred male compared to female urine, supporting the findings of previous studies signifying the importance of chemical communication in female mate choice in bank voles^28–31,36^ and consistent with female preference for male urine in other vole species^37–39^. In our study, females spent more time investigating both male and female urine compared to a water control but, when not actively investigating, were motivated to spend more time only near male urine. This contrasts with a recent study in water voles where females spent more time investigating male urine versus a water control but not female urine^37^. Female bank voles are solitary, occupy exclusive home ranges, and typically avoid other females^33^, which may explain their motivation to investigate scent from other females but reluctance to spend time near this stimulus.

In our study airborne urinary VOCs from both sexes attracted female bank voles to investigate the scent. However, attraction was much stronger to male stimuli, and females only spent more time nearby scents that were from males when not actively investigating, suggesting that male urinary VOCs play an important role in sexual attraction in bank voles. This contrasts with female attraction to urinary scent signals in house mice and rats, where females display similar levels of approach and investigation to male and female airborne VOCs unless they have learned sexual attraction to a particular individual male odour profile in association with MUP pheromones^5,6,16,40^. Although we did not conclusively identify any male urinary VOCs in this study, preliminary analysis suggests that the urinary volatiles profile of male bank voles is dominated by a single peak. A recent study in water voles showed female attraction to a male urinary volatile, 3-ethyl-2,5-dimethyl-pyrazine, when females were presented with a pure version of this compound^41^. Further work is needed to confirm the identity of the major peak present in male bank vole urine and whether it is attractive to females.

In bank voles, females are strongly attracted to airborne VOCs without needing further information from an unfamiliar male’s scent. Attraction to airborne volatiles without prior exposure suggests that attraction to male urinary VOCs is a general response to male odour, rather than to the scent of a particular individual male as has been demonstrated in mice^18^. Male bank voles occupy overlapping home ranges and females do not select mates based on their ability to defend a high-quality territory^33^, so there may be less need for male bank voles to signal individual identity to females than in territorial species^16^. Male and female bank voles are solitary and occupy relatively large home ranges, so one role of urinary VOCs could be to bring opposite sex conspecifics together, rather than a mechanism for assessing and selecting competing mates.

Although females were strongly attracted to male scents when contact was prevented, this does not mean that scent components detected on contact do not play an important role in sexual attraction in bank voles. When females were presented with only LMW components from male urine, females showed clear attraction when they could not contact the source but only minimal attraction with contact. While airborne VOCs are important for attracting females to approach and investigate male scent, females clearly gain additional information influencing sexual attraction on scent contact.

Female bank voles were attracted to the protein component of male scent, but we found no evidence that the male-specific OBP, glareosin, induced female attraction. In our study, females were tested with a fraction of male urine containing glareosin, it is possible this fraction also contained other minor components below the level of detection. However, the presence of these components in the fraction used to test females does not alter the fact that we found no evidence for female attraction to a normal physiological level of glareosin when it was added to non-breeding male urine. The sex-specific and seasonal expression of glareosin suggest its function relates to male reproductive behaviour^22^. In house mice, female sexual attraction to male urine is driven by the male-specific MUP, darcin^18^. Although darcin binds male volatile pheromones, the protein alone can induce attraction in females^16^. In contrast scent OBPs may require bound ligands to be biologically active. Aphrodisin, a hamster vaginal OBP, only induces sexual behaviours when bound by its natural ligands^17^. Similarly, salivary lipocalin 1 (SAL1), an OBP expressed in the saliva of domestic pigs, binds the male volatile pheromones 5α-androst-16-en-3α-ol and 5α-androst-16-en-3-one that cause females to adopt a mating posture^42,43^. Whether or not glareosin binds ligands present in male urine is currently unknown, but it is possible that any bound ligands may have been removed during the process of protein purification and/or storage, potentially explaining why the addition of purified glareosin did not increase female attraction.

Alternatively the main function of high investment in glareosin by males during the breeding season may be to greatly extend the duration of volatile signals that attract females to investigate and spend time near the male’s scent^22^. In house mice, after the initial loss of unbound ligands that are highly volatile, urine that contains a high concentration of MUPs retains a higher concentration of volatile ligands and releases these over a longer time period^10,11,44^. Although we did not specifically test for bound glareosin ligands, female attraction to male HMW fraction when contact was prevented, even after scents had been aged for 5 days, provides strong evidence that proteins within male urine bind VOCs.

It was surprising that females did not show increased chemosensory responses to breeding compared to non-breeding male urine, as has been demonstrated in other vole species^38^. In our study, the failure to detect a significant difference in response to urine collected in different seasons was driven by a greater than expected response to non-breeding male urine, rather than a reduction in response to urine from breeding males. This suggests that males may excrete VOCs throughout the year, which our study shows are attractive to females. Currently there is limited understanding of variation in urinary volatiles between the breeding and non-breeding seasons, despite observations in other vole species indicating seasonal changes in urinary volatile profiles^21^. Due to experimental constraints, our breeding males originated from a captive colony, while non-breeding males were wild-caught. Male signals are often an indicator of male quality and female attraction to male signals can be condition dependent^45^. Captive males' VOCs and urinary protein levels might not reflect those of a high quality male, potentially reducing female responses. In a prior study, males from our captive bank vole colony had male-biased urinary protein expression similar to wild individuals in the breeding season, but wild males generally exhibited higher mean protein output than captive-bred ones^22^. Nevertheless, captive-bred males still had higher mean protein output than males caught in the non-breeding season^22^.

Overall, this study adds to the existing body of work demonstrating the importance of chemical signals and cues in facilitating mammalian reproductive behaviours. Although the precise mechanism of attraction is yet to be determined in bank voles, our results suggest it differs from those previously described in murid rodents. In particular, the greater attraction to male versus female urinary volatiles without prior contact, and the lack of attraction to the male-specific protein glareosin is consistent with diverse functions of urinary proteins between species. Further studies investigating female responses to male scents in a much wider range of species are needed to explore commonalities in female attraction.

## Methods

### Subjects

Subjects were 24 adult female bank voles, *Myodes glareolus* (8 wild-caught and 16 captive-bred). Wild caught animals were captured as adults from three different populations in the northwest of England, at least four months prior to the start of the experiments. Captive bred animals were F1 generation aged 3–10 months from a captive colony at the Mammalian Behaviour and Evolution Group, University of Liverpool, UK. Prior to the experiment, females were singly housed in plastic cages (43 × 11.5 × 12 cm). All animals were maintained under controlled environmental conditions, with temperature 20–21 °C, relative humidity 45–65%, and a reversed 16 h light:8 h dark photoperiod representative of the breeding season (lights off at 09:00). All animals were provided with ad libitum access to water and food (Lab Diet 5LF2 Certified Rodent Diet; Purina Mills, St. Louis, MO), supplemented with commercially available parakeet mix (various suppliers), vegetables (baby corn or carrot) and fruit (apple, blackberries, blueberries, or raspberries), placed in their home cages twice a week. Subjects were housed on Lignocel® wood fibres substrate with paper wool nest material. Cardboard tubes and boxes were provided for enrichment. Captive bred females were sexually mature at the time of testing but had not bred. The sexual experience of wild caught females was unknown. Prior to testing all subjects were exposed to male and female conspecifics and their scent (supplementary methods). Some females had been used in a previous experiment investigating female competition and scent marking behaviour.

### Behavioural assays

Behavioural tests were carried out in enclosures (70 × 60 × 55 cm). A thin layer of Lignocel® substrate covered the floor of enclosures. Subjects were singly housed in testing enclosures for two weeks prior to the start of the experiment, to allow them to familiarise with the set up as their home area, and remained in enclosures for the entire experimental period. Nesting material and cardboard enrichment were provided to females but removed during behavioural tests. All testing was carried out during the dark phase of the light cycle. In all tests subjects were presented with two stimuli at the same time. Stimuli were presented on glass microfibre (GMF) paper (5.5 cm diameter) attached to the centre of a Benchkote-covered Perspex tile (15 × 15 × 0.5 cm) using double sided sticky tape. Stimuli were streaked onto GMF paper to replicate the shape of a male bank vole scent mark. All stimuli were left to dry for 5 min before presentation to subjects.

Female responses to conspecific urine were investigated by presenting subjects with their own urine alongside one of three test stimuli: male urine, female urine, or MilliQ-grade H_2_O. Four 5 µL streaks of urine or water were streaked onto GMF paper. Stimuli were presented either covered by a mesh cap (diameter 5 cm), to prevent subjects from directly contacting the urine whilst still allowing airborne volatiles to be detected, or uncovered so both airborne volatiles and non-volatile compounds could be detected.

To investigate female attraction to different components of male urine, subjects were presented with a male urinary fraction, separated by molecular weight, alongside the equivalent female urinary fraction. Urine was fractionated into high and low molecular components using 3 kDa cut-off centrifugal filters (supplementary methods). The high molecular weight (HMW) fraction should contain no more than 2% of the original low molecular weight material in intact urine that is not bound to protein. The low molecular weight (LMW) fraction could contain smaller peptides < 3 kDa, typically less than 25 amino acids in length. Female subjects were presented with male LMW or HMW fractions presented alongside the equivalent female fraction. Subjects were also tested with male and female HMW fractions, presented at the same time, that had been aged for five days to allow VOCs to evaporate. Fresh HMW fraction was streaked onto GMF paper and left uncovered at ambient temperature for 5 days. Two 20 µL streaks of urinary fractions were streaked onto the GMF. All stimuli were presented either covered by a mesh cap or uncovered to explore the effect of direct contact on female responses. The LMW and HMW fractions from the same male sample were used to test different females on the same testing day. The order of testing was randomised where possible, but the LMW fraction was used immediately after fractionation and before the matching HMW fraction to minimise the loss of volatile compounds.

Female response to glareosin was investigated by presenting subjects with male urine collected in the non-breeding season alongside non-breeding male urine with 1.5 µg/µL of glareosin added, in a two-way preference test. Glareosin was purified from mature male bank vole urine using liquid chromatography (LC) (supplementary methods). Previous studies investigating male urinary protein expression indicate that 1.5 µg/µL of protein falls within the range of normal protein expression for mature adult males^22^. In a second test, subjects were presented with male urine collected from a captive colony housed under breeding season conditions and male urine collected outside the breeding season. Stimuli were presented by streaking two 20 µL streaks of urine from a male in the breeding season, non-breeding season or non-breeding season with glareosin added, onto the GMF paper. Stimuli were presented uncovered in all tests allowing subjects to contact the stimuli.

In all tests, tiles were placed on either side of the enclosure 9 cm away from the enclosure walls. This allowed plenty of space for females to move around the walls of the enclosures without having to contact tiles. The position of the two tiles was randomized but balanced to ensure an equivalent number of each stimulus was presented on each side. The order subjects received each of the stimuli and the order subjects were tested in each day was randomised. Clean nitrile gloves were worn when handling tiles to reduce any transfer of scent from the experimenter to tiles. Subjects were removed from their enclosure while bedding and enrichment were removed, and scent stimuli introduced. Subjects were re-introduced at the centre of the enclosure, equally distanced from both tiles, using plastic tunnels to avoid directly handling the subject. Equivalent tiles lined with Benchkote and mesh caps, when used, were placed in the females’ home enclosures during the two-week familiarisation period. Clean tiles and caps were used in all behavioural tests. All trials lasted 20 min and were recorded remotely from an adjoining room. At least 48 h was left between trials using the same subject. Individual females were used in a single experiment for a maximum of 6 trials.

### Urine donors

Urine donor bank voles were 33 males (17 captive bred (F1–3), aged 3–10 months and 16 wild caught) and 20 females (10 captive bred (F1), aged 3–10 months, and 10 wild caught). Wild caught individuals were captured during the breeding season at least two weeks prior to testing, brought into the lab and housed under the same conditions as captive animals. Non-breeding male urine samples were collected from 5 wild caught males, (wild voles caught in late November, normal breeding season March–October). Captive bred voles were derived from the same colony as subjects but were unfamiliar and unrelated to them (individuals did not share a common ancestor in the previous two generations). Scent donors were housed singly in plastic cages (43 × 11.5 × 12 cm) under the same conditions as female subjects, except for non-breeding males which were housed outside under shelter in plastic cages (43 × 11.5 × 12 cm) under non-breeding season temperatures (range 4 °C–15 °C) and light conditions (number of daylight hours: 8.0–9.5).

Urine samples were collected by isolating each male vole in a clean clear plastic cage (40 × 24 × 12 cm). Males were placed on a metal grid over the cage and a second plastic cage (40 × 24 × 12 cm) was inverted over the grid and secured using clips. The bottom cage was made of clear plastic allowing urine to be easily identified while reducing disturbance of urine donors. Voles were checked regularly and returned to their home cage after urination or after a maximum 2 h if they failed to urinate. Urine was collected from the bottom of cages using a pipette, transferred to 1.5 mL Eppendorf tubes and stored at -20 °C. Subjects were tested with urine from a different donor in each test and the specific donor was randomised. At least 48 h were left between sample collections from the same individual. Urine was collected under red light conditions during the dark period from all individuals except for non-breeding males where urine was collected during daylight hours.

### Behavioural analysis

Female behavioural responses were transcribed from video recordings of behavioural tests using BORIS software ^46^. All videos were blinded for subject, stimuli, and tile side to reduce experimenter bias during analysis. It was not possible to blind videos for whether subjects could contact the scent, as the presence or absence of a mesh cap could be seen in the video footage.

To assess female attraction to a stimulus, we measured the amount of time females spent sniffing a stimulus and the amount of time they spent near the stimulus when not sniffing. Sniffing was defined as when females place their nose directly onto or very close to (within 3 cm) the GMF paper or mesh cap. The total time females spent on the tile, including the time spent sniffing stimuli, was also recorded. Females were considered on a tile when all four feet were on the tile. We calculated the amount of time females spent on the tile when not sniffing the stimulus by subtracting the time spent sniffing from the total time spent on the tile.

### Statistical analysis

Statistical analysis was completed using RStudio, with R version 4.2.0^47,48^. We used linear mixed effect models (LMM) to investigate female attraction and female investigation of conspecific urine and urinary fractions. All LMM were fitted using the package lme4, version 1.1.29^49^. P-values for fixed effects were generated using the Anova function of the car package, version 3.0.13^50^. All LMM were validated using Q/Q and residual plots. Where variables were found to violate model assumptions, a transformation was applied, and the residuals rechecked. The Akaike Information Criteria (AIC) was used for model selection and non-significant interactions were removed from final models. Where multiple models were within 2 AIC units of the best supported model, the most parsimonious model (with the fewest parameters) was chosen.

All figures were produced using the R packages ggplot2, version 3.4.0^51^, gridExtra, version 2.3^52^ and cowplot, version 1.1.1^53^.

#### Female attraction to male urine

To assess if female attraction to male urine was greater than to female urine to or water, we calculated bias scores by subtracting the amount of time females spent near or sniffing their own urine from time spent near or sniffing test stimuli. We modelled the bias towards test stimuli for either the time females spent sniffing the stimulus or nearby not sniffing, against the fixed effects: stimulus (male urine, female urine, water) and contact (contact or no contact). We also tested for a significant interaction between the fixed effects stimulus and contact. Female ID was included as a random effect to account for repeat testing of the same female. As bias scores for both time sniffing stimulus and time near the stimulus did not meet model assumptions, a ln(s + 1) transformation was applied to the time sniffing and time nearby each stimulus and the bias scores were re-calculated using transformed variables. Planned comparisons were used to test if females spent more time sniffing or near male urine compared to female urine or water. Planned comparisons were fitted using the emmeans package, version 1.7.4.1^54^, with a Kenwards Roger degree of freedom approximation. Post hoc contrasts were used to test for differences in female responses between female urine and water, also conducted using the emmeans package. One female did not visit either own urine or test tiles in 4 out of 6 trials so all her data was removed from the final analysis due to failure to interact reliably with the test.

#### Female attraction to male urinary fractions

To determine the importance of non-volatile urinary components in stimulating female attraction, we compared female responses to LMW and HMW urinary fractions. First, we checked that females were more attracted to male compared to female urinary fractions by modelling the amount of time females spent either sniffing or near a stimulus against donor sex (male or female), urinary fraction (HMW vs LMW or HMW vs aged HMW) and contact (contact or no contact). We nested trial within female ID as a random effect to control for repeat testing of females and to compare male and female fractions presented in the same test. A ln(s + 1) transformation was applied to time spent sniffing and time nearby stimuli.

To determine if female preference for male urinary fractions was dependent upon the fraction presented or whether females could contact the scent, we calculated the bias towards the male stimulus. Bias to male stimulus was calculated by subtracting the time spent sniffing or nearby not sniffing the female from the equivalent male fraction. We modelled bias to male stimulus against urinary fraction (fresh HMW vs LMW or HMW vs aged HMW) and contact (contact or no contact). Again we checked for a significant interaction between fraction and contact. Where the final model included a significant interaction, we use planned comparisons to test if females responses to a specific fraction (LMW, HMW or aged HMW) were greater when the could contact the scent compared to when contact was prevented. Planned comparisons were fitted using the emmeans package, version 1.7.4.1^54^, with a Kenwards Roger degree of freedom approximation. We included female ID as a random effect to account for repeat testing of females. A ln (s + 1) transformation was successful in meeting normality assumptions for time nearby and time sniffing in HMW vs aged HMW models, confirmed by residual plots and Shapiro-Wilks tests. However, this was not the case for time sniffing stimuli in the LMW vs fresh HMW model where a square root transformation was required. No transformation was required for the time spent nearby stimuli in the HMW vs LMW model, as raw values successful met normality assumptions. One video file was corrupted for a single HMW contact trial and is therefore not included in any of the analyses.

#### Female attraction to glareosin

To determine if female attraction to male urine was stimulated by glareosin we used paired t-tests, performed using the R stats package. We first tested if females spent a greater amount of time nearby or sniffing breeding male compared to non-breeding male urine. We then tested for differences in the time females spent nearby or sniffing non-breeding male urine spiked with glareosin compared to non-breeding male urine. A ln(s + 1) transformation was successful in meeting normality assumptions for residuals (confirmed by Shapiro-Wilks tests and Q/Q plots) for time spent nearby the stimulus. However, this was not the case for time sniffing where a square root transformation was required, with Q/Q plots and Shapiro–Wilk normality test used to confirm that transformed data fitted test assumptions.

### Ethical statement

All procedures involved in this study were non-invasive behavioural tests. Animal use and care was in accordance with the UK Home Office code of practice for the housing and care of animals bred, supplied, or used for scientific purposes and EU directive 2010/63/EU. All experiments reported in this study were carried out in compliance with the ARRIVE guidelines. The University of Liverpool Animal Welfare Committee approved the work, but no specific licenses were required.

### Supplementary Information

Supplementary Information.

## Data Availability

All data and code to re-create the results and figures can be found in the Figshare Digital Repository (https://figshare.com/s/25dd1f13a73408ff8b34).
