# Supplementary material for: The role of male scent in female attraction in the bank vole, Myodes glareolus
Source: Sci Rep. 2024 Feb 27;14:4812. doi: 10.1038/s41598-024-55235-x (PMC10899570; doi:10.1038/s41598-024-55235-x)
Supplement: Supplementary file 1 — Supplementary Information. [file 41598_2024_55235_MOESM1_ESM.docx]

# **Supplementary Information**

## Supplementary methods

### Exposure to conspecifics and their scent

In the wild, bank voles are solitary but will regularly encounter the scents of conspecifics, and occasional directly interactions. As individuals in our captive colony are singly housed to match the solitary behaviour of wild voles, they have limited experience of contact with unrelated conspecifics or their scents. Therefore, to replicate a more natural social experience, all female subjects were exposed to some conspecifics and their scents prior to any behavioural tests. As previous studies have shown that novelty can influence behavioural responses in rodents ^[1,2]^, prior exposure to some conspecific scents reduced the likelihood that female responses were simply due to the presence of a novel stimulus.

Females were exposed to the scent of two unrelated, unfamiliar male and female conspecifics from the same captive colony as female subjects, deposited naturally on soiled paper nest material. Bedding from one female and one male was introduced three weeks prior to the start of the experiment when females were housed in home cages. A week later bedding from a second male and female was introduced when females were housed in experimental enclosures where behavioural tests were carried out. All bedding was replaced with clean bedding one week before the start of the experiment, to ensure that only the conspecific scent being tested was present during behavioural trials.

We also carried out short interactions between female subjects and male and female conspecifics. These interactions were carried out in addition to the bedding transfers, to ensure female subjects had been exposed to a full range of social stimuli prior to testing. Two weeks prior to the experiment, females underwent five-minute encounters with unfamiliar, unrelated male and female conspecifics. These short encounters were conducted in a clean enclosure. Females were introduced into the enclosure and allowed to explore the environment for five minutes, after which either a male or female conspecific was introduced. Two days later the process was repeated with the second conspecific. Half of the females received a male conspecific first and half received a female conspecific first. Two items of clear plastic enrichment were placed in the enclosure during these interactions to provide cover. Interactions were monitored via a video camera from an adjoining room, with an ethical rule in place to halt interactions if mating or continuous fighting or chasing occurred for more than 10 seconds. No interactions had to be halted due to aggression.

During all behavioural trials, females were tested with an unfamiliar donor whose scent they had not previously encountered either through direct interactions or via soiled bedding.

### Urine fractionation

Urine was separated into high molecular weight (HMW) and low molecular weight (LMW) components using Vivaspin 500 centrifugal concentrators (Vivascience), with a 3 kDa molecular weight cut-off. As a single urine sample is typically less than 500 µL, multiple samples from the same individual were pooled prior to fractionation. Vivaspins were first washed twice using 500 µL MilliQ-grade H2O centrifuged at 15,000g (according to manufacturer recommendations) for 15 minutes, emptied of residual water, 500 µL of urine was added and centrifuged at 15,000g for 15 minutes. The material passing through the filter was referred to as the ‘LMW fraction’. The retentate in the Vivaspin was subsequently replenished with water to the original volume, and centrifuged once more, to deplete the HMW fraction of further LMW material that was not protein bound – this second pass through fraction was discarded. We estimate that this process reduced loosely associated LMW material to less than 2% of the original. Material retained in the filter was referred to as the ‘HMW fraction’. Both LMW and HMW fractions were pipetted into clean 500 µL Eppendorf tubes and used immediately. The second LMW fraction was not used in any behavioural tests.

Following behavioural tests, the HMW and LMW fractions were stored at ‑20°C. To confirm that fractionation had been carried out successfully, the HMW fractions, LMW fractions and intact urine (samples from the same individual pooled prior to fractionation) were analysed for protein concentration and composition. Total protein concentration was determined using the Pierce Coomassie plus protein assay reagent kit (Thermo Fisher Scientific). Bovine serum albumin (BSA) was used as a standard and initially diluted to generate standards in the range of 0 - 30 μgmL^‑1^. To ensure samples were within the linear range of the assay, urine samples were diluted with MilliQ-grade H2O. Absorbance was measured at 620 nm using a microplate photometer (Thermo Multiskan FC Microplate Photometer, Thermo Fischer Scientific). A linear standard curve was generated from the absorbance readings of the BSA standards, from which the protein concentration of unknowns was calculated. If sample absorption fell above the BSA standard absorption range, samples were re-analysed using BSA standards in the range of 0 - 50 μgmL^-1^.

HMW fractions, LMW fractions and pooled intact urine were also analysed by SDS-PAGE, performed as described Laemmli ^[3]^. All intact and fractionated urine samples were mixed 1:1 with reducing sample buffer (100 mM DTT) and then heated for 5 minutes at 100°C. Samples were loaded so that 15 µL of sample and buffer solution was loaded per well. All samples were run at a constant voltage of 200 V on 15% SDS gels. Protein bands were visualized with Coomassie brilliant blue stain.

### Loss of volatiles from aged male bank vole urine

To assess how the volatile composition of male urinary scent marks changed over time, we conducted a preliminary analysis of the volatile composition on male bank vole urine. To explore differences between male and female urinary volatiles, urine was collected, from 2 captive-bred female (F1-2), aged 3-6 months, and 2 captive-bred males (F1-2), aged 4-7 months. Urine was analysed by GC-MS by extracting 50 µL of freshly defrosted urine with 200 µL hexane (GC grade, Sigma-Aldrich), in 2 mL sealed glass vials topped with aluminium lined caps (Supelco), for 2 hours at room temperature (18-24°C). After extraction 30 µL of the hexane layer was transferred to a sealed 300 µL interlock vial for analysis by GC-MS. Solvent aliquots (1 µL of the hexane extract) were analysed using a GCT Premier Mass Spectrometer (Waters, Manchester, UK), fitted with a VF-5HT 30 m x 0.25 mm internal diameter x 0.1 µm film thickness capillary column (Agilent J&W, Santa Clara, CA, USA). The initial oven temperature was 40^o^C and elevated at 12^o^C min^-1^ to 260^o^C. The splitless injector mode was used, and injector temperature was maintained at 250^o^C. Helium was used as the carrier gas at a constant flow of 0.8 mLmin^-1^. The mass spectrometer (MS) inlet line was maintained at 270°C and the source temperature was 180°C. The ionisation energy was 70 eV and the trap current was 200 mA. The mass spectrometer scanned the mass range *m/z* 40 – 500 Da with a scan time of 0.5 seconds, following a 4-minute solvent delay. Mass spectra were then searched against the NIST (National Institute of Standards and Technology) mass spectral library to identify known peaks.

We also conducted a preliminary GC-MS analysis to assess loss of volatile composition of male urinary scent marks over time since deposition. Freshly defrosted male urine was streaked onto GMF paper in 50 µL spots and left to dry for 0, 0.5, 1, 2, 4 and 24 h at ambient room temperature (18 - 24°C). The same urine sample from a captive-bred F1 male bank vole (aged 4 months) was used for all time points. Dried spots were cut into small pieces and extracted with 200 µL of hexane (GC grade, Sigma-Aldrich) in 2mL sealed glass vials, topped with an aluminium lined cap (Supelco). For the 0 hour sample, 50 µL of male urine was added to pieces of GMF in a vial and hexane extraction began immediately. Solvent aliquots (1 µL of the hexane extract) were analysed using the same instrument and methods described above.

As high intensity peak with a retention time of 16.8 minutes dominated the volatile profile of fresh male urine (Fig S1), we measured the total ion current (TIC) area of this peak using the integrated peaks function in Mass Lynx (Waters, Manchester, UK). All peaks in the chromatogram showed an overall decline with time since deposition, with no volatile peaks visible from 24-hour aged urine (Fig S2). After 30 minutes, the peak at 16.8 minutes had declined by over 50% compared to fresh urine and was not detectable in urine that had been aged for 24 hours (Fig S3).


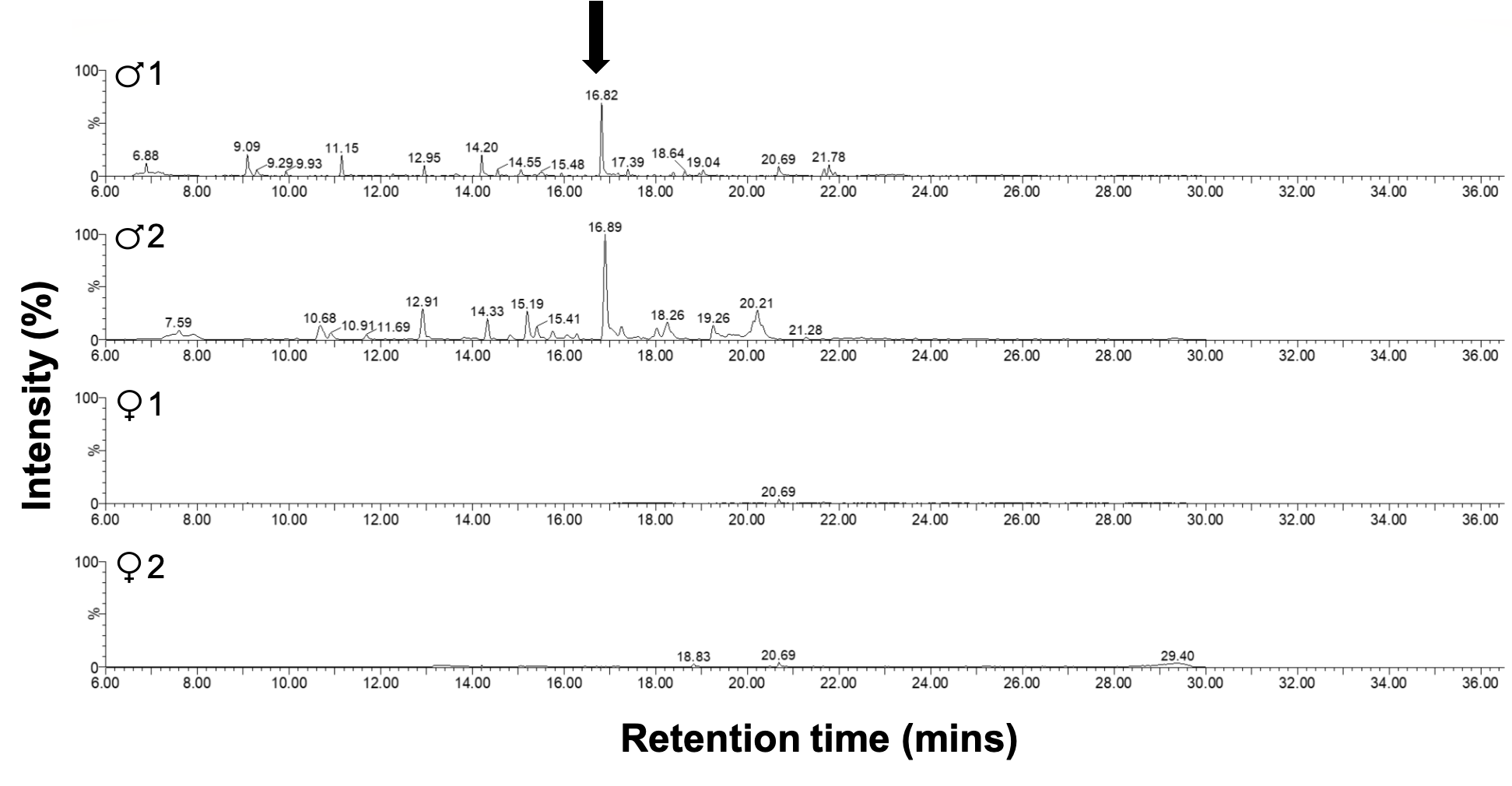


Supplementary Figure S1. Urine collected from male and female bank voles was analysed by gas chromatography mass spectrometry. Chromatograms are presented on the same scale for comparison. Black arrow indicates the major peak at 16.82 minutes.

###
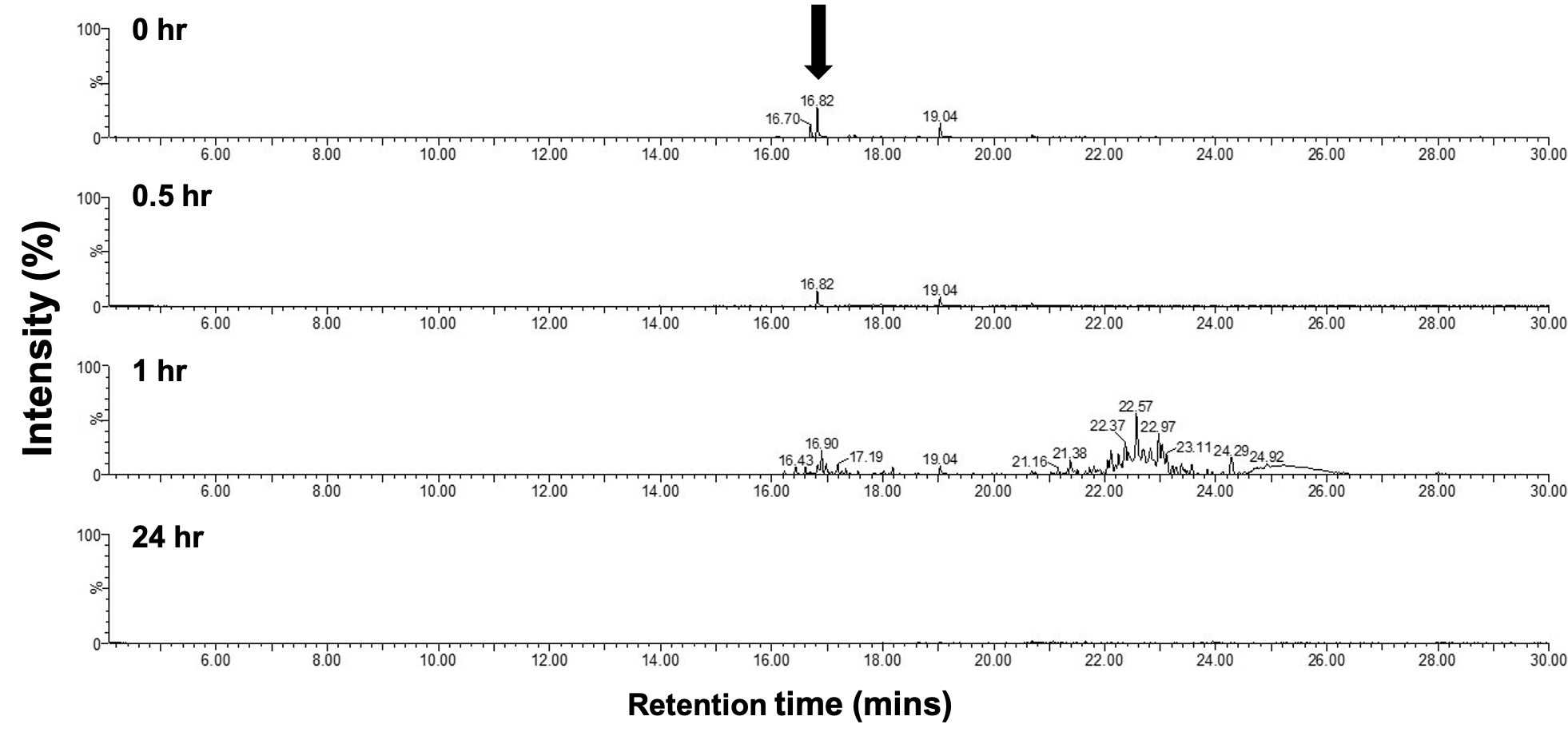


Supplementary Figure S2. Male urine was aged at ambient temperature for 0, 0.5, 1 and 24 hours and analysed by gas chromatography mass spectrometry. All chromatograms are presented on the same scale for comparison. The same urine sample was used for all four time points. Black arrow indicates the major peak at 16.82 minutes.

###
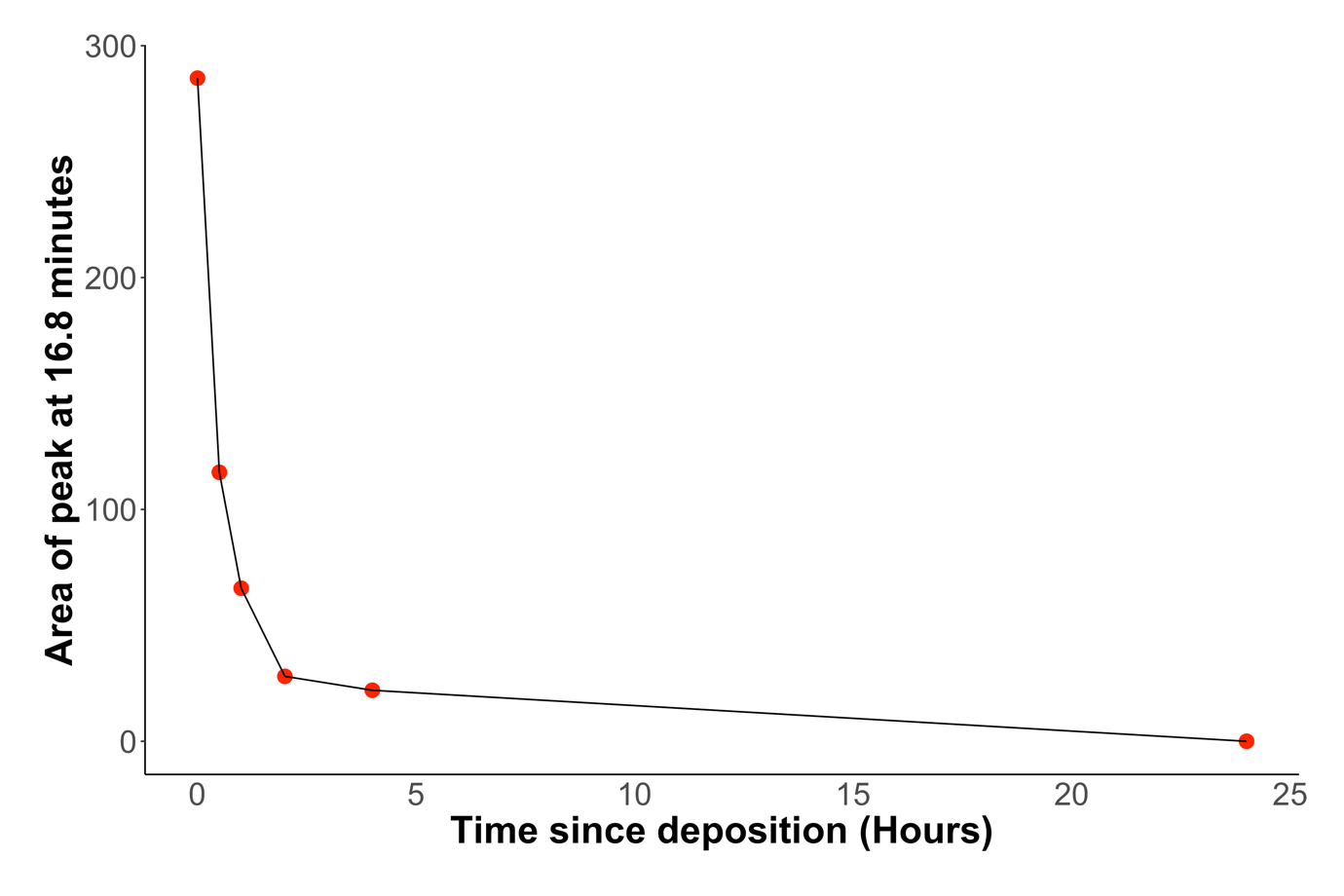


Supplementary Figure S3. Male urine was aged at ambient temperature for 0, 0.5, 1, 2, 4 and 24 hours and analysed by gas chromatography mass spectrometry. Total ion current peak areas of the peak at 16.8 minutes were calculated using the integrated peaks function in Mass Lynx (Waters, Manchester, UK). The same urine sample was used for all time points.

### Glareosin purification

Glareosin was purified from mature male bank vole urine using anion exchange liquid chromatography (LC). Urine samples were collected from 16 captive bred male bank voles (F1 - 2), aged 3 - 10 months. Captive bred voles were derived from the same colony and housed under the same conditions as female subjects. Urine was collected under red light conditions following the same method described for urine donors. Individual males were sampled multiple times over a period of 17 days, with a maximum of 2 samples collected during a 7 day period. Purification of glareosin from intact male urine was carried out on an ÄKTA purifier liquid ResourceQTM column (GE Healthcare Lifesciences, V = 1 mL). Prior to purification the column was equilibrated in 20 column volumes of 10 mM HEPES buffer, pH 8.0 at 1 mLmin-1. Urine samples from multiple males were pooled prior to purification and 500 µL of desalted, pooled urine was injected into the column. Bound protein was eluted from the column using a linear salt gradient of 0 - 1M NaCl in 10 mM HEPES, pH 8.0, over 20 column volumes at 1 mLmin^-1^. A total of 9 fractions were manually collected as individual peaks following a real-time UV trace (Fig S4).

SDS-PAGE and electrospray ionisation mass spectrometry (ESI-MS) were used to identify which of the nine LC fractions contained glareosin. SDS-PAGE was carried out following the same method as described for urinary fractions. Fraction 4 contained a single band that matched the corresponding band in intact male urine identified as glareosin (Fig S5). Prior to ESI-MS fractions were desalted by adding 100 µL of LC fraction to a 500 µL Zeba column (Thermo Fischer Scientific) with a 7 kDa molecular weight cut off. Prior to use Zeba columns were centrifuged at 15,000g for 1 minute to remove storage buffer. Zeba columns were first washed twice using 500 µL MilliQ-grade H2O centrifuged at 15,000g for 2 minutes, emptied of residual water, LC fraction was added and centrifuged at 15,000g for 2 minutes. The desalted fractions were diluted 1 in 100 in 95% water / 5% acetonitrile / 0.1% formic acid. Diluted samples were run on a Waters’ Synapt G2-Si mass spectrometer in positive ion mode coupled to a Acquity HPLC system fitted with a with a MassPREP micro desalting column. The injection volume was 1 µl and protein was eluted at a flow rate of 25 µLmin^-1.^ Three repeated 8-minute gradients of 95% 0.1% formic acid in water, 5% 0.1% formic acid in acetonitrile to 10% 0.1% formic acid in water and 90% 0.1% formic acid in acetonitrile were used to release the protein from the column. The instrument was calibrated with 0.5 µL of 250 fmol equine heart myoglobin (Sigma-Aldrich). Spectra were acquired over the mass range *m/z* 50 to 2,000 Da. Multiply charged spectra were de-convoluted using MaxEnt1 (Waters) over the range 16 to 18 kDa at resolution 0.25 Da/channel. Fraction 4 contained a major peak at 16,929 ± 1 Da (Fig S6) which matches, within instrument accuracy, the predicted molecular weight of glareosin of 16,930 Da ^[4]^. Therefore, fraction 4 was used as glareosin in all behavioural tests.

The protein concentration of fraction 4 containing glareosin was determined with the Coomassie plus protein assay reagent kit following the same method described above for urinary fractions. Prior to behavioural tests glareosin fractions were concentrated using Vivaspin 500 centrifugal concentrators (3 kDa molecular weight cut-off) to a concentration of 1.5 µgµL^-1^.

## References

1. Cavigelli, S. A., Michael, K. C., West, S. G. & Klein, L. C. Behavioral responses to physical vs. social novelty in male and female laboratory rats. *Behavioural Processes* **88**, 56–59 (2011).
2. Hughes, R. N. Neotic preferences in laboratory rodents: Issues, assessment and substrates. *Neurosci Biobehav Rev* **31**, 441–464 (2007)
3. Laemmli, U. K. Cleavage of structural proteins during the assembly of the head of bacteriophage T4. *Nature* **227**, 680–685 (1970).
4. Loxley, G. M. *et al.* Glareosin: A novel sexually dimorphic urinary lipocalin in the bank vole, *Myodes glareolus*. *Open. Biol.* **7**, 170135 (2017).

| 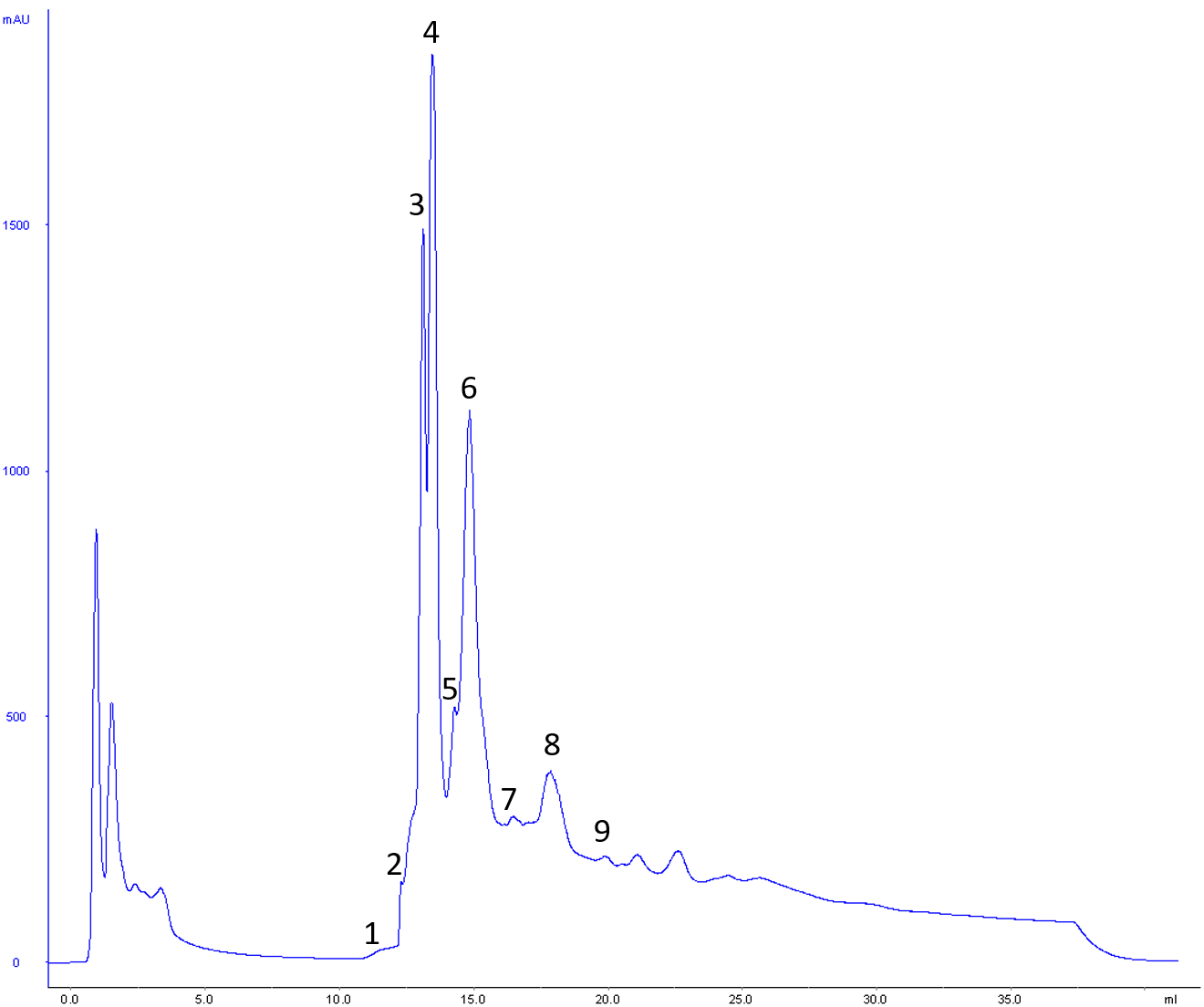 **Absorption**  **Retention time (mins)** |
| --- |
| Supplementary Figure S4: Nine fractions were recovered from liquid chromatography fractionation of urine from adult male bank voles. Fraciton 4 represents the fraction later identified as glareosin that was used in behavioural tests. |
| Supplementary Figure S5. Nine liquid chromatography fractions were analysed by SDS-PAGE for the presence of glareosin. Lanes from left to right, LC fractions 9 - 1, Mk: marker. Fraction 4 shows a band at ~17 kDa which would include glareosin (16,930 Da). Intact male urine from fig 3d included for comparison and to highlight migration of glareosin, full gel included as fig S7. |


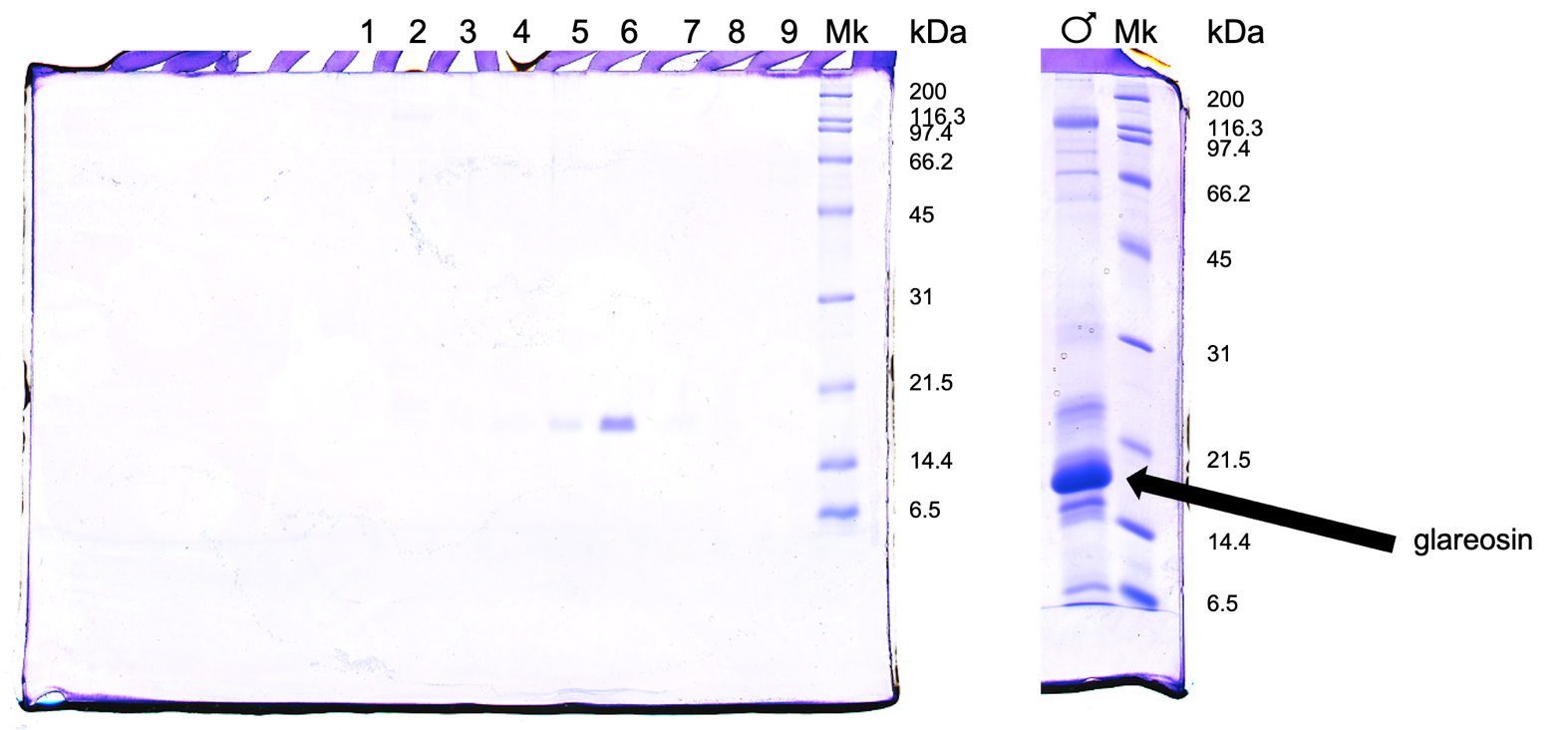


Supplementary Figure S6. Fractions from liquid chromatography were analysed by intact mass spectrometry. Fraction 4 contained a single main peak at 16,929 ± 1 Da which is within instrument accuracy of the predicted molecular weight of glareosin of 16,930 Da.


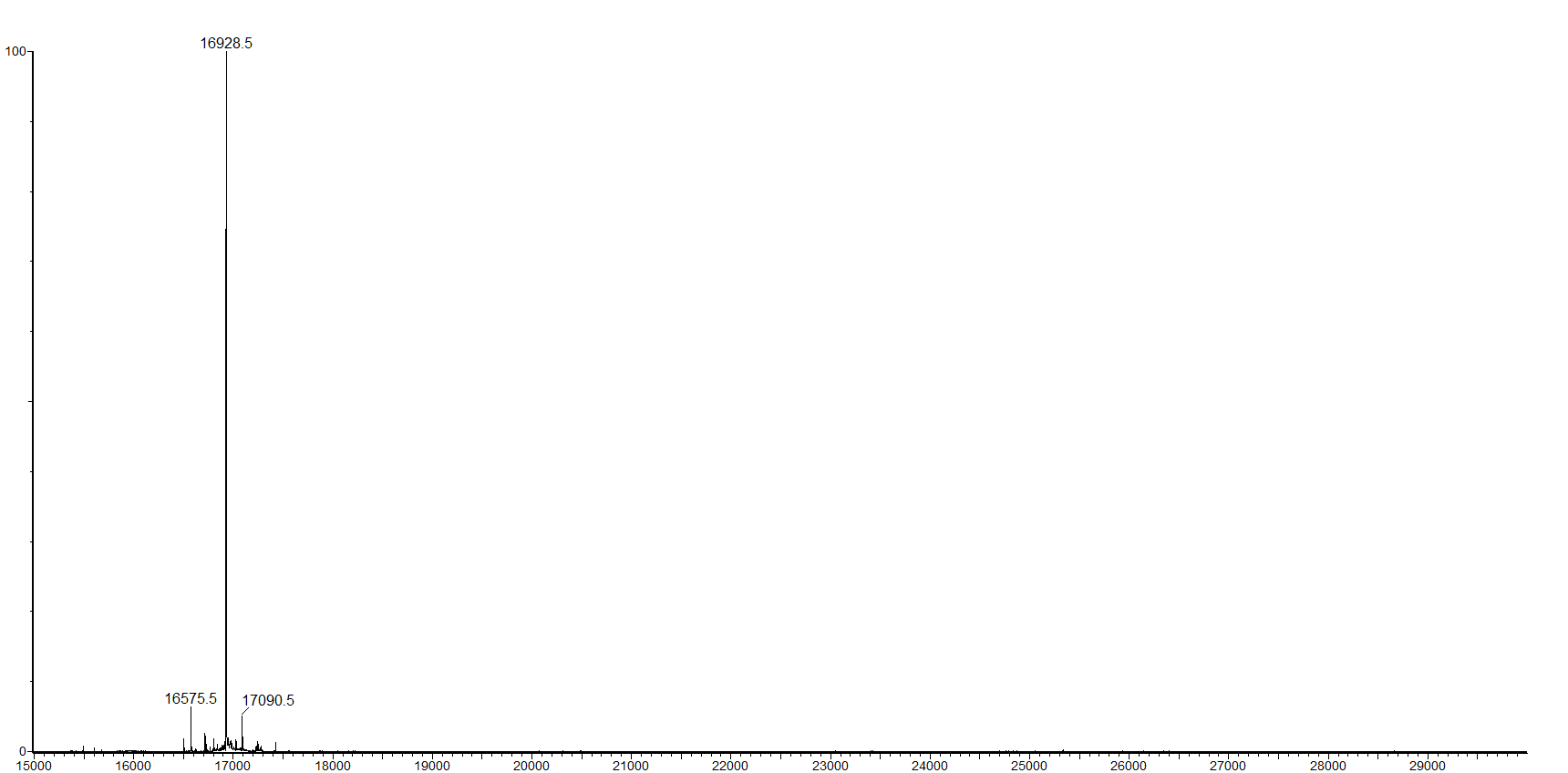


**Relative intensity (%)**

**Mass (Da)**


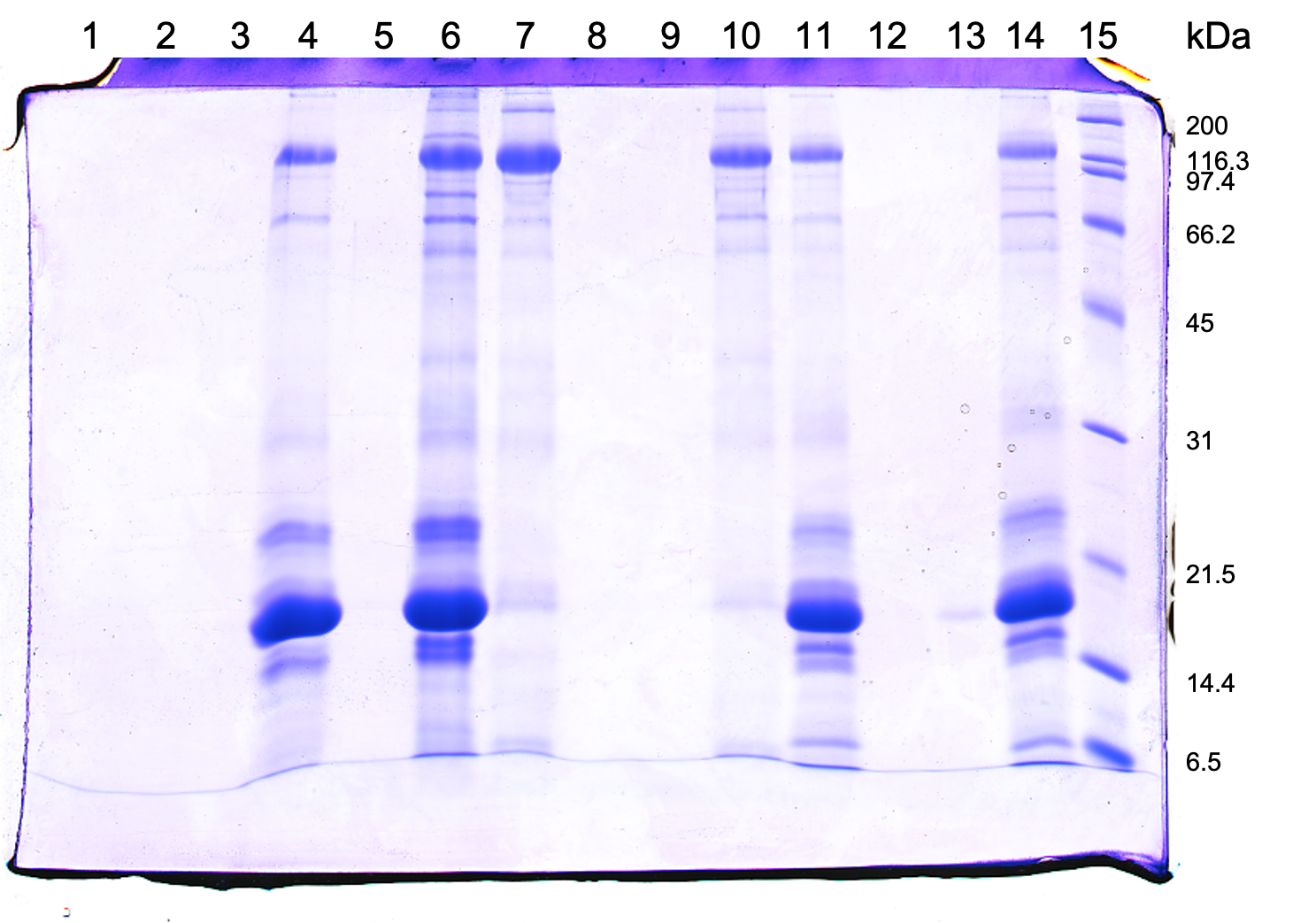


Supplementary Figure S7. Original SDS-PAGE image for male bands included in figure 3d. Wells 11 - 14 included in fig 3d. From left to right (1) empty well, (2) empty well, (3) male 1 LMW 2, (4) male 1 HMW, (5) male 1 LMW 1, (6) male 1 intact urine, (7) female 1 HMW (8) female 1 LMW 2, (9) female 1 LMW 1, (10) female 1 intact urine, (11) male 2 HMW, (12) male 2 LMW 2, (13) male 2 LMW 1, (14) male 2 intact, (15) marker.


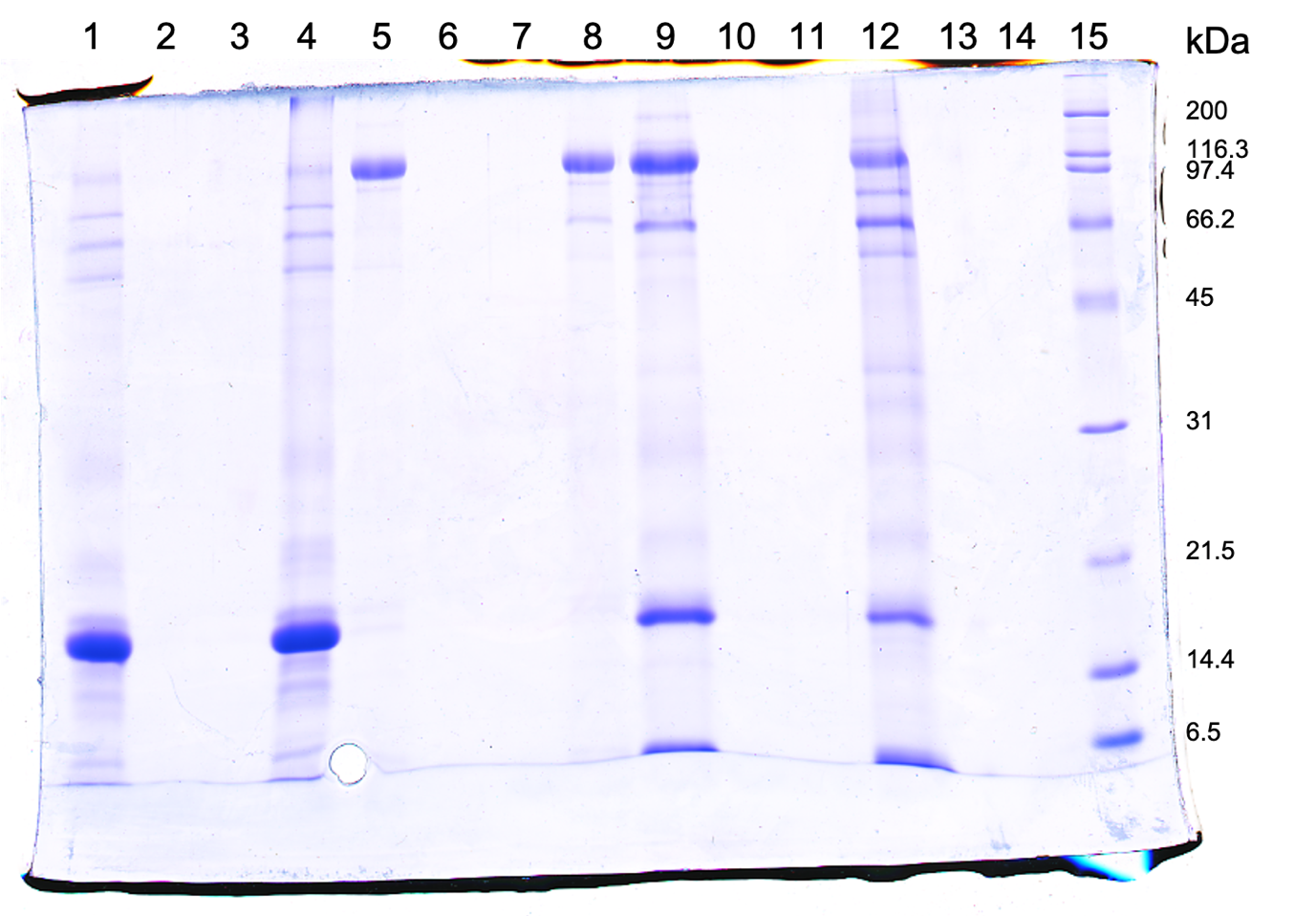


Supplementary Figure S8. Original SDS-PAGE image for female bands included in figure 3d. Wells 5 - 8 and 15 included in fig 3d. From left to right (1) male 3 HMW, (2) male 3 LMW 2, (3) male 3 LMW 1, (4) male 3 intact urine, (5) female 2 HMW, (6) female 2 LMW 2, (7) female 2 LMW 1 (8) female 2 intact urine, (9) male 4 HMW, (10) male 4 LMW 2, (11) male 4 LMW 1, (12) male 4 intact urine, (13) empty well, (14) empty well, (15) marker.


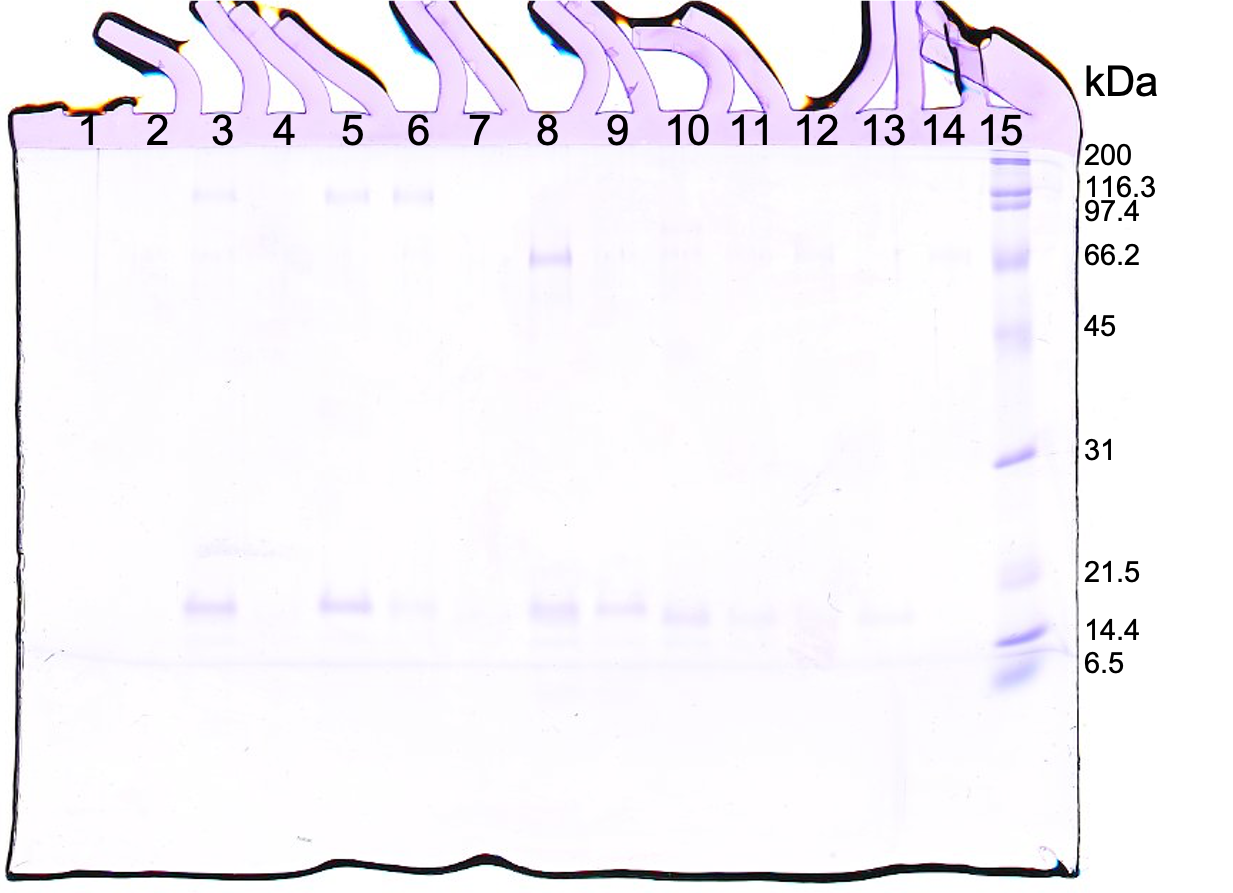


Supplementary Figure S9. Original SDS-PAGE image for non-breeding male urine included in fig 4e. Wells 1 – 4 included in fig 4e. Urine samples were collected from wild-caught non-breeding males housed in outdoor enclosures during the non-breeding season. Urine samples were collected November – December, non-breeding season November – February. From left to right (1) non-breeding male 1, (2) non-breeding male 2, (3) non-breeding male 3, (4) non-breeding male 4, (5) – (14) samples from another experiment unrelated to experiments or data presented here (15) marker.


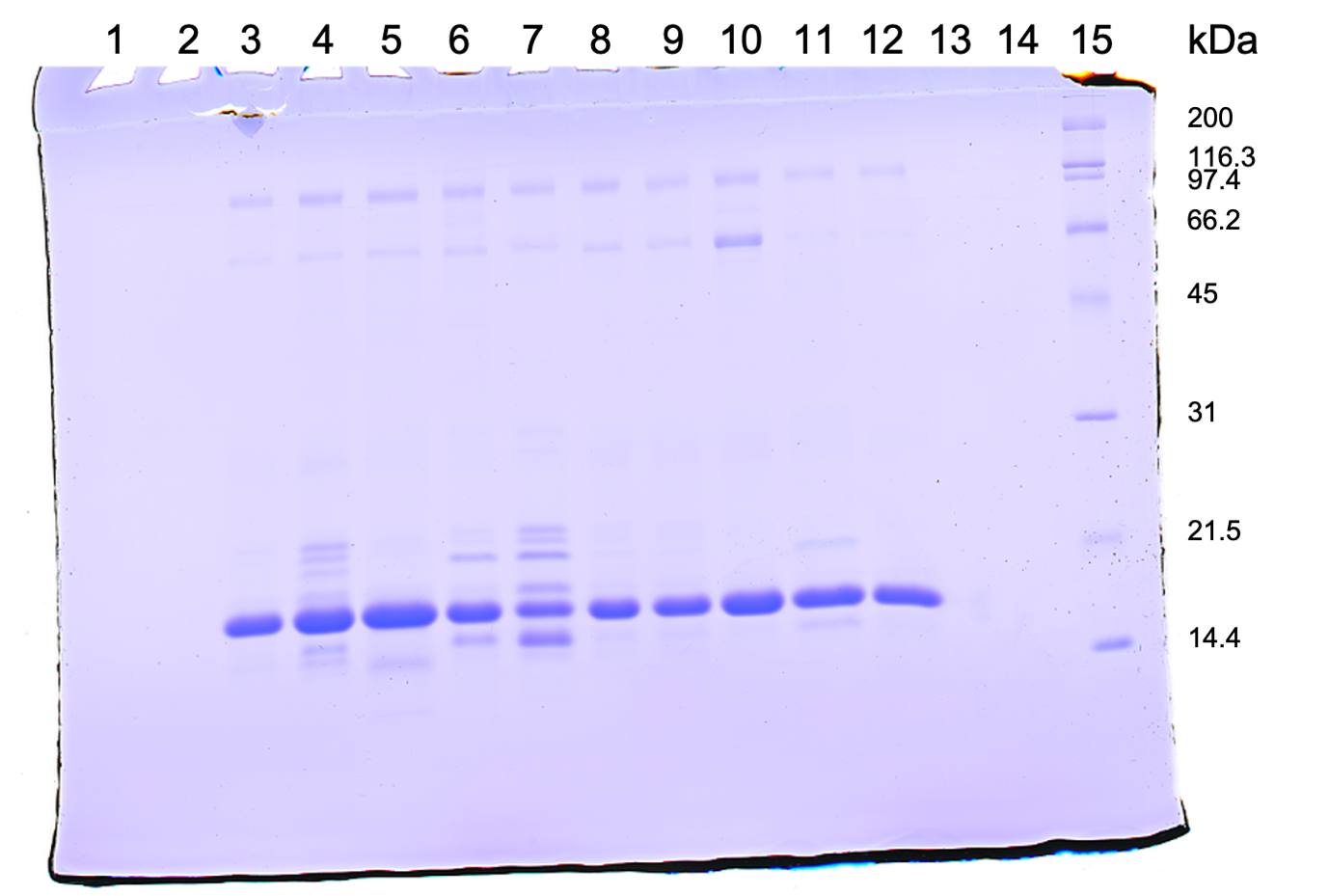


Supplementary Figure S10. Original SDS-PAGE image for breeding male urine included in figure 4e. Wells 12 – 8 included in fig 4e. Urine samples were collected from adult male bank voles from our captive colony housed under breeding season conditions during November – December, which equates to the non-breeding season in wild voles. From left to right (1) empty well, (2) empty well, (3) male 1 sample 1, (4) male 1 sample 2, (5) male 1 sample 3, (6) male 2 sample 1, (7) male 2 sample 2, (8) male 3 sample 1, (9) male 3 sample 2, (10) male 3 sample 3, (11) male 3 sample 4, (12) male 4 sample 1, (13) empty well, (14) empty well, (15) marker.
